# Applications of Electronic Nose, Electronic Eye and Electronic Tongue in Quality, Safety and Shelf Life of Meat and Meat Products: A Review

**DOI:** 10.3390/s23020672

**Published:** 2023-01-06

**Authors:** Paulo E. S. Munekata, Sarah Finardi, Carolina Krebs de Souza, Caroline Meinert, Mirian Pateiro, Tuany Gabriela Hoffmann, Rubén Domínguez, Sávio Leandro Bertoli, Manoj Kumar, José M. Lorenzo

**Affiliations:** 1 Centro Tecnológico de la Carne de Galicia, Rúa Galicia N° 4, Parque Tecnológico de Galicia, San Cibrao das Viñas, 32900 Ourense, Spain; 2 Food Preservation & Innovation Laboratory, Department of Chemical Engineering, University of Blumenau, 3250 São Paulo St., Blumenau 89030-000, Brazil; 3 Department of Horticultural Engineering, Leibniz Institute for Agricultural Engineering and Bioeconomy, 14469 Potsdam, Germany; 4 Chemical and Biochemical Processing Division, ICAR–Central Institute for Research on Cotton Technology, Mumbai 400019, India; 5 Facultade de Ciencias, Universidade de Vigo, Área de Tecnoloxía dos Alimentos, 32004 Ourense, Spain

**Keywords:** quality control, shelf life, safety, adulteration, preservation, processing

## Abstract

The quality and shelf life of meat and meat products are key factors that are usually evaluated by complex and laborious protocols and intricate sensory methods. Devices with attractive characteristics (fast reading, portability, and relatively low operational costs) that facilitate the measurement of meat and meat products characteristics are of great value. This review aims to provide an overview of the fundamentals of electronic nose (E-nose), eye (E-eye), and tongue (E-tongue), data preprocessing, chemometrics, the application in the evaluation of quality and shelf life of meat and meat products, and advantages and disadvantages related to these electronic systems. E-nose is the most versatile technology among all three electronic systems and comprises applications to distinguish the application of different preservation methods (chilling vs. frozen, for instance), processing conditions (especially temperature and time), detect adulteration (meat from different species), and the monitoring of shelf life. Emerging applications include the detection of pathogenic microorganisms using E-nose. E-tongue is another relevant technology to determine adulteration, processing conditions, and to monitor shelf life. Finally, E-eye has been providing accurate measuring of color evaluation and grade marbling levels in fresh meat. However, advances are necessary to obtain information that are more related to industrial conditions. Advances to include industrial scenarios (cut sorting in continuous processing, for instance) are of great value.

## 1. Introduction

Meat is a key source of nutrients due to its content of protein, fat, iron, zinc, niacin, and vitamins B6 and B12 [1]. Unfortunately, meat is easily degraded if it is not properly handled or preserved, causing serious health risks to consumers and economic loss for producers [2]. In meat degradation, microbial deterioration and biochemical reactions have an important role, once improper storage accelerates these reactions [3]. As a result, carbohydrates, proteins and fats are decomposed into acetaldehyde, hydrogen sulfide and ammonia by the action of enzymes and bacteria, producing different types of gases during the meat degradation process [4].

In refrigerated foods (usually recommended at 4 °C), the progression of food spoilage is reduced and may be difficult to detect the end of shelf life [5,6]. In terms of quality and processing control, physicochemical measurements are usually obtained (such as pH, color, and concentration of chemicals) for food authentication, and sensory analysis is fundamental for the evaluation of attributes to establish an organoleptic profile of various products [7]. Traditionally, these analyses are costly, last for long periods, and are carried out by trained personnel or specialized staff (especially for sensory analysis) in laboratory infrastructure. Some examples are microbial analysis lasting for at least many days; chemical and chromatography methods requiring continuous expenses with reagents, gases, supplies, and expensive equipment; and sensory analysis (a subjective evaluation) that involves many panelists to judge the acceptance of selected attributes. Another key aspect of these methods is the destruction of samples, hindering further analysis [8,9].

Alternative solutions have been studied and developed to obtain economic and fast protocols with easy implementation (portability, for instance) for industrial applications and advances for industrial applications, especially for the current 4th Industrial Revolution [8,10]. This scenario has been supporting the development of E-nose, E-eye, and E-tongue to evaluate the flavor, aroma and appearance of several foods in an objective way [11]. These technologies were created based on the working principles of the human senses by aiming to analyze, recognize, discriminate and predict the quality of foods [12].

The use of electronic tools to evaluate the quality of food and improve our knowledge about their properties has been studied in several foods. For instance, the use of E-nose assisted in the indication of origin in differentiation of Chinese Yunnan coffee from different locations and also indicated that the variation among samples can be attributed to coffee roasting, grinding, and brewing [13]. Another interesting application of electrochemical tools (E-tongue system) is the discrimination of olive oils in terms of processing conditions (particularly for malaxation temperature) [14]. The equipment had similar predictive capacity for correct classification than a trained panel to distinguish samples in terms of malaxation temperature.

Other interesting applications include the use of E-nose for the classification of carrots in terms of surface defects (presence of bad spots, abnormalities, and formation of fibrous roots) [15] and the discrimination of cheese according to aging period [16], E-eye to detect adulteration of black peppercorns with papaya seeds [17], and E-tongue to identify adulterated wine [18]. All these studies support the emerging role of electronic tools in several lines of research related to food quality.

Although previous reviews have summarized the use and applications of electronic devices mimicking human senses in fresh meat or covering a specific device [19,20,21], a review about the key aspects covering both fresh meat and meat products with information about each current technology in this field is still needed. Therefore, this review aims to synthesize the information about the fundamentals of E-nose, E-eye, and E-tongue, data preprocessing, key chemometric methods (to manage and obtain key information), the wide range of applications to evaluate both fresh meat and meat products, relevant advantages and disadvantages to devices, and recommendations for further studies.

## 2. E-Nose System

**Figure 1:**
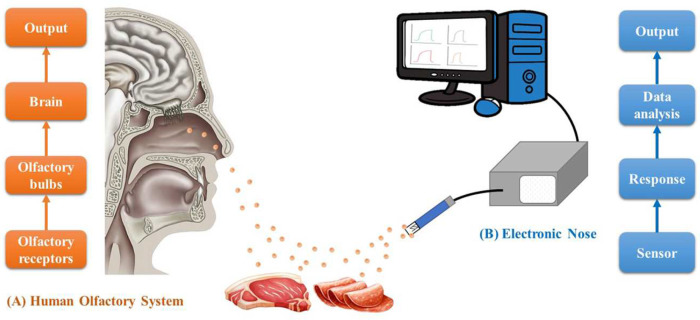
Schematic illustration of the biological olfactory system (**A**) and E-nose technology (**B**).

The E-nose is an odor analysis instrument that mimics the mammalian olfactory system [2]. This technology comprises a large range of electrochemical sensors for pattern recognition, capable of recognizing simple and complex odors [22]. This electronic instrument is characterized by a multisensory technology, dating back to the 1960s, when the first mechanical instruments for simulating human olfactory capabilities were developed, and have received several improvements in their concept and functioning since then [23]. Basically, the E-nose system consists of a sampling system (handle and storage samples during analysis), sensor array (collect signals from the interaction of sensing material with different volatile compounds), and computer (data storage, pre-processing and processing) [24,25,26], as presented in Figure 1.

The sensor array can be composed of many field-effect metal-oxide-semiconductor transistors with catalytic metal action gates or chemical sensors based on carbon dioxide brass, such as the so-called Taguchi sensors [24]. Additionally, it is possible to use E-nose for detection of toxic gases, which disqualifies the human nose as a universal classification tool [27].

### Principle of Odor Sensors

According to Karakaya et al. [28], in an E-nose, the electronic sensor array corresponds to the olfactory receptors in the human nose, which detects traces of chemicals present in the air. The volatile molecules are detected by the receptors in the nose and the signal is sent to the olfactory bulb, which processes the odor into a stimulus for recognition in the brain, as presented in Figure 1. In its electrochemical counterpart, E-nose components are the hardware and software, where the software can be considered as the “brain”, and the hardware (set of sensors) is considered as the “olfactory receptors”. The sensors interact with volatile compounds and generate a signal pattern that will then be processed and classified using the digital signatures of the detected chemicals [28].

Comparatively, about 100 million sensors are involved in the perception of odors in humans, while in the E-nose, the perception is carried out by the array of sensors. Thus, the recognition function of the olfactory bulb is replaced by a computer that extracts the features and pattern recognition using an algorithm [2]. In other words, the extensively tuned E-nose chemical sensors interact with the volatile compounds from the sample and generate an electrical signal that is processed by an appropriate chemometric tool [7].

Sensors are composed by different materials, such as metal-oxide-semiconductor (MOS), field-effect metal-oxide-semiconductor transistor sensors (MOSFET), mass sensitive sensors, conductive organic polymers (CP), solid electrolyte sensors (SES), and optic fiber sensors, have been the most prevalent, as well as acoustic wave chemical vapor sensors with ultrasonic frequencies mainly from 1 to 500 MHz [23]. MOS sensors are widely used and have a successful commercial basis. The detection mechanism of MOS sensors is based on the change of conductivity of the oxide in the presence of an oxidant or reducing gas by reduction/oxidation reactions taking place on the surface of the sensor [25].

One company producing E-nose equipment is Airsense (www.airsense.com, accessed on 20 July 2022) that is based in Schwerin, Germany, (founded in 1996) and produces the PEN3 model. The company states that this model can be used to evaluate freshness, presence of off-odors, residual solvents, and aromas in foods. This portable model is built with 10 sensors that can provide readings after 1 min at temperatures between 0 and 45 °C, relative humidity in the range of 5–95%, and sample flow between 10 and 400 mL/min. This system can be connected to a computer for data processing [29]. Another company producing E-tongue systems is Sensigent (www.sensigent.com, accessed on 16 December 2022). This company is located in Baldwin Park, California, (USA) and produces the Cyranose 320. This E-nose is composed of 32 nanocomposite sensors to measure volatile compounds at ppm level. The system is portable and is compatible with different algorithms (such as Hierarchical Clustering Analysis) [30]. The E-nose systems market also comprises companies in different countries. One example is Sacmi (www.sacmi.com, accessed on 16 December 2022) that is located in Italy and produces the EOS 912 system [31]. Food Sniffer^®^ is a commercial, portable E-nose system that connects to a smartphone and indicates, after few seconds, the condition of the food (fresh, starting to spoil, or spoiled). This sensor is produced by FoodSniffer (http://www.myfoodsniffer.com/, accessed on 16 December 2022), which is based in Redwood City, USA, and can be used by final consumers [32].

## 3. E-Eye System

Color is a fundamental component in food quality, since it is closely linked to the perception of freshness, ripeness, desirability and food safety, and is often considered by consumers when buying food [33]. In other words, color is the mental perceptual response to the visible spectrum of light reflected or emitted from an object. This response is achieved from the interaction between light and receptors located on the retina, which generates a stimulus that is directed to the brain by the optic nerve. The perception of color is dependent not only on the object but also on the environmental illumination [34]. For this reason, color analysis is extremely important for the classification of products such as meat and cultivated foods such as peas, corn, canola, rice and wheat for human and animal consumption [33].

The E-eye is a detection technology based on recognition and analysis of visual information applied to food quality evaluation [35]. The human eye is able to distinguish colors by differentiating wavelengths. Color spaces vary according to sensitivity based on the hue saturation (HS) space, such as HSI (hue, saturation, intensity), HSV (hue, saturation, value), HSL (hue, saturation, lightness) and HSB (hue, saturation, brightness) [34].

**Figure 2:**
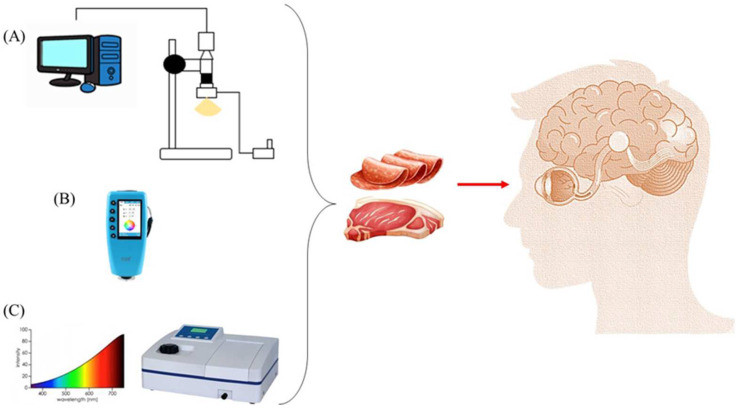
E-eye’s Operating System: (**A**) Computer Vision, (**B**) Colorimeter, (**C**) Spectrophotometer.

The E-eye has favorable characteristics for its application in food quality evaluation such as being cost-effective, portable and easy to apply on a large scale [36] This technology can be based on colorimetry, spectrophotometry or computer vision (Figure 2). The choice of the appropriate equipment can assure a more effective color description [25].

Thus, E-eye is able to capture the appearance-related characteristics of samples, but cannot detect the flavor or aroma-related components, and for this reason, the combination of multiple technologies such as E-nose and E-tongue has become an alternative to detect the various characteristics of samples [37].

### Principle of Visual Sensors

The sensors and color identification techniques fall into three methods based on colorimetry, spectrophotometry and computer vision. For the food industry, the most widely used color space system is the L*a*b*, also known as the CIELAB system, first defined by the CIE in 1976, where L* is a measure of the lightness of the sample color, a* indicates red or green colors, and b* coordinate is used for yellow and blue colors [33].

Similar to the CIE, the human eye has three color receptors, red, green and blue, and all their possible combinations. Thus, the quantities of each, used for the formation of any and all colors, are called tri-stimulus values and are denoted X, Y and Z, respectively, and were created in 1931 by the International Commission on Illumination [38]. Additionally, colorimeters are capable of measuring primary and secondary sources of radiation which emit and reflect light, respectively, in order to simulate an observer’s response, requiring an illumination source, a combination of filters, and a photoelectric detector [38].

Spectrophotometers are technologies for measuring color by the spectral distribution of transmittance or reflectance of a sample, which provides a spectral analysis of the reflectance wavelength and/or transmission properties of objects [38]. Near-infrared reflectance spectrophotometers (NIR) are also used in the food industry to measure chemical constituents such as proteins, oil, starch, fiber and moisture to assess the quality of products where color is critical, e.g., meat [33].

Computer vision is another color measurement technology, originating in the 1960s, with applications ranging from medical diagnostic imaging to remote sensing [39]. The functioning of this equipment is based on algorithms for automatic extraction and analysis of information from a set or sequence of images of an object in a fast, consistent, non-invasive and cost-effective way [34]. However, as the image size increases, algorithms become proportionally more complex and the processing time increases [40].

Furthermore, computer vision emerged to overcome the deficiencies of colorimeters, due to its ability to determine color readings for each pixel of an image, and has wide application for color measurement of animal origin foods [41]. The high spatial resolution of computer vision allows analysis of each pixel to calculate the mean and standard deviation of the color and characterize the appearance by extracting data from a region of interest while generating a map of the color distribution in a sample [34].

Typically, computer vision systems are based on a connection of electrical and mechanical devices in which illumination is an important prerequisite for image evaluation and determination of product quality, which reduces the time, complexity, and the cost (in some cases) of image processing steps [40]. Recent advances in hardware and software have contributed to the expansion of computer vision systems in the food industry [39], which is ranked among the top 10 industries using this technology. Some viable image processing hardware can be implemented such as application-specific integrated circuits (ASICs), digital signal processors (DSPs), and field programmable gate arrays (FPGAs) [40].

One example of a company producing visual sensors is Loccus Biotecnologia (www.loccus.com.br, accessed on 20 July 2022) located in Cotia, Brazil (founded in 2003). Loccus produced the Doc L-Pix image benchtop system that is customizable with CCD progressive scan of scientific grade, resolution 3.1 megapixels, exposition times between 1 ms and 60 s, color filters, motorized optical zoom, and illuminator with selectable wavelength. The equipment can be connected to a computer system for data management [42]. One relevant type of equipment that can assist in the electronic measurement of color and shape of foods is the IRIS Smart Vision, commercialized by Alpha M.O.S (www.alpha-mos.com, accessed on 20 July 2022) and based in France (founded in 1992). This model is equipped with a high resolution camera mounted in a closed cabinet [43].

## 4. E-Tongue Tongue System

Taste is the perception associated with the presence of substances that stimulates taste buds located on the tongue [6]. The E-tongue, unlike the human tongue, has improvements in sensitivity, selectivity and multiplexing capacity of modern biosensors that are able to reliably predict the quality of samples quickly and accurately, earning space in the pharmaceutical, environmental, cosmetics and food industries [44].

**Figure 3:**
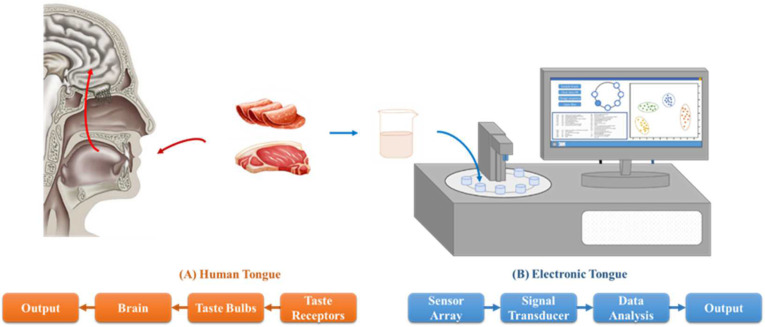
Schematic illustration of the biological taste system (**A**) and E-tongue technology (**B**).

The term E-tongue appeared in the 1990s, an analogy to the human tongue, and has been intensively researched with new articles being published every year [12]. It is a technology composed of a set of sensors that react when immersed in chemical solutions (Figure 3). Basically, it consists of an electrochemical cell (sensor array), a measurement module and an appropriate pattern recognition capable of recognizing simple and complex systems of molecules that form the taste [6].

Thus, the electronic tongue extracts a signal signature from a sample by analyzing its individual components [45]. E-tongue can characterize the taste of a complex liquid or samples converted to liquid form [46]. The main goal of this technology is the analysis of foods by a set of sensors (such as ion-selective electrodes with specific properties) followed by statistical analysis. Consequently, information about freshness and level of maturity can be obtained [46,47].

The sensor array of an E-tongue generates multidimensional information in a short period of time; this technology recognizes the different patterns of classes of compounds responsible for the flavor. The complex information generated from E-tongue measurement is then processed with multivariate statistical analysis [7]. In comparison, the human taste and flavor perception process also comprises the match of signals from taste buds with human memory to define the flavor and taste [46]. In addition, a wide variety of chemical sensors can be used for the sensor suite of an E-tongue such as electrochemical, optical, mass and enzymatic [7].

### Principle of Taste Sensors

Taste has a fundamental role in determining the quality of food. By definition, basic tastes are separated according to taste perception, where salinity indicates electrolyte balance in food; acidity indicates decomposition; bitterness prevents the ingestion of poisonous materials; umami informs the presence of amino acids; and sweetness is able to indicate nutritional sources [6]. The E-tongue starts from the working principle of the human tongue. By using non-selective chemical sensors capable of detecting specific chemical substance, it responds globally to the non-volatile compounds present in the sample in order to recognize taste patterns (Figure 3) [45].

These sensory taste results can be processed using numerical methods and visualized in a plot [12]. There are several models of chemical sensors based on taste patterns used to design the set of electronic sensors that form the E-tongue. These sensors are classified into electrochemical (potentiometric, voltammetric, amperometric, impedimetric, and conductimetric), optical, mass, and enzymatic biosensors [12,20].

Potentiometric sensors were the first and are still the most widely used for E-tongue applications due to advantages such as low cost, simple assembly, and manufacturing, and are the closest resemblance to the natural taste recognition mechanism [7]. From this group of sensors, ion selective electrodes (ISE) are the most widely used [46]. The potential is measured by two electrodes in the absence of current flow to determine the concentration of a component in the analyzed solution, based on the measurement of changes in its potential against the reference electrode [12,48].

Some disadvantages of using this type of sensor are temperature dependence, influence of changes in solution composition, and the adsorption of components (affecting charge transfer) [12]. Taste measurement occurs by submerging the reference electrode in an electrolytic solution where the electrode potential (E) is determined as a function of the analyte concentration of the ratio of the oxidized ($C_{o}$) and the reduced form ($C_{r}$), which can be expressed by the Nernst equation (Equation (1)). *E* is the electrode potential at standard conditions, T is the temperature [27], F is the Faraday constant, and η is the amount in mol of the electrons transferred.

(1) $$E=E_{0}+\frac{RT}{\eta F\left(ln\frac{C_{o}}{C_{r}}\right)}$$

The Equation (1) indicates the reduction potential of an electrochemical reaction, depending on the standard electrode potential, temperature, and the activities of the chemical species in the oxidation-reduction process; serving as a basic model for the sensor response and as a basis for classical models; and avoiding mathematical, numerical and computational difficulties arising from the solution of nonlinear problems in advanced models [49].

Unlike the potentiometric model, voltammetric measurement is used when there is no equilibrium. For the measurement, three electrodes are required: a constant flow reference electrode, a working electrode, and an auxiliary current flow electrode [12]. Furthermore, in the volumetric technique, a potential is applied to the working electrode, after which the resulting current between the working electrode and the reference electrode is measured, resulting in an electrochemical redox reaction that occurs at the surface of the electrodes and leads to the measurement [7].

Chalcogen glass sensors are also used for E-tongue, can be applied in various analytical problems, and be coupled with ion-selective polyvinyl chloride membrane electrodes [12]. This membrane can be lipid/polymeric (composed of a lipid, polyvinyl chloride, and a plasticizer) and used in the receiving stage of the taste substances (thickness of about 200 μm) for about 3000 readings [6]. The taste sensor, using a lipid polymer membrane, has been developed to respond to the taste of chemical substances and can be used to characterize the taste based on the five basic tastes, specifically bitter, sour, sweet, salty, and umami (determined using individual solutions containing quinine, acetic acid, sucrose, sodium chloride, and monosodium glutamate, respectively) [47].

The perception of each basic taste is essential to evaluate the sensory quality of meat and meat products. In fresh meat, the basic tastes have been indicated as main taste descriptors regardless of species (such as veal, cow, and bull), quality grade (high vs. low), aging process (dry- vs. wet-aging), and packaging system (vacuum-packaged, modified atmosphere, or wrapping) [50]. Meat products can also be sensorially described by all basic tastes, although some variations in the selection of basic taste descriptors may be observed due to the type of product and processing conditions. This aspect can be observed in studies with dry fermented pork loin [51], dry-aged beef [52], and sausages [53].

One of the commercial E-tongues available in the market is the α-Astree model produced by Alpha M.O.S. This model is composed of an autosampler detection system with 7 sensors and a computer system for processing data. The detection system is composed of organic membranes that can interact with ionic, neutral, and organic compounds at room temperature with readings every 200 s [43]. Insent (www.insentjp.com, accessed on 16 December 2022) is another company that manufactures E-tongue systems. Some of the models produced by this company, located in Japan, are the SA402B and TS-500Z [54].

## 5. Data Pretreatment

Acquiring useful data from measurements with E-nose, E-eye, and E-tongue can be a challenging task due to the presence of unwanted variations in the data set that can affect the quality of data. The low quality of data can be attributed to different factors that include missing data (values outside the instrument range or equipment malfunctioning), noise (non-properly calibrated sensors), base line shift (continuous background signals), peak shifts (unexpected variations in experimental conditions) [55,56]. This challenging scenario lead to the development of data preprocessing methods and strategies to preserve relevant information and remove unwanted variation before the application of chemometric data [55].

Current studies indicate that preprocessing techniques for E-nose data varies among studies and highlights the importance of considering the evaluation of data in a case-by-case basis. For instance, the studies carried out with fresh meat using PEN3 nose indicate the necessity to correct sample data using blank samples’ sampling results [57,58] and the removal of outliers from the experimental dataset [59]. In the case of E-nose systems assembled by the authors of the study, the corrections of baseline and data normalization have been reported for studies with both fresh meat [1] and meat products [60].

The raw data obtained from E-eye measurement also must be properly pretreated. In general, the first step consists in defining the Region of Interest (ROI) by removing the unnecessary information from the acquired image. Essentially, a greyscale conversion followed by binarization of image can be applied to separate background region from ROI [61,62,63]. It is also possible to erode the images to enhance the differences at the boundary of these two regions prior to binarization [64,65,66]. Additionally, it may be necessary to perform manual adjustments such as reported by Teimouri et al. [67], who indicated that manual removal of small blobs were necessary to remove areas incorrectly indicated as ROI.

It is also important that some adjustments can be applied to the image prior to the identification of ROI. This consideration can be observed in the study carried out by Geronimo et al. [65] with chicken breast affected by wooden breast myopathy (white stripes aligned among the muscle fibers), which applied the normalized illumination of images prior to the identification of ROI. This strategy reduced the effect of incident light spots and facilitated the segmentation of white striations.

Once the ROI is identified, specific strategies can be applied to enhance the quality of data, especially for the segmentation of areas of intramuscular fat in both fresh meat and meat products. This process can be carried out using color space blue [68] or red [61], or other strategies such as the Sobel image processing method [63], contrast limited adaptive histogram equalization [65] and Fourier transform [69]. Particularly for texture segmentation, Sun et al. [63] used a gray-level co-occurrence matrix in images obtained from fresh pork loin.

Particularly for the application of E-tongue in meat and meat products, one of the possible strategies to deal with voltammetry data was indicated by Apetrei and Apetrei [70]. These authors pretreated data obtained from fresh beef using an E-tongue system with a protocol composed of the following sequential steps: separation voltammograms into anodic or cathodic, multiplication by a factor of 10 to smooth data, bell-shaped windowing functions, and integrating according to the potential. Considering the complexity of datasets obtained from E-nose, E-eyes, and E-tongue, and the necessity to have high-quality data for chemometric analysis, it seems reasonable to highlight a single and universal method to obtain a high-quality data set, as well as the number of methods or their sequence, regardless of electronic system. This condition also suggests that data pretreatment should be performed considering the intended application (evaluation of quality decay during shelf life, identification of intramuscular fat, or color evaluation, for instance) rather than following a standard protocol.

## 6. Chemometrics Methods

The use of electronic systems in the evaluation of meat and meat product characteristics generates complex and large datasets that require adequate data analysis to extract relevant information. Chemometrics is a key strategy to comprehensively manage datasets by using data-driven methods that reveal relationships and create mathematical models to characterize measured properties and variables of interest [71,72]. Chemometric methods can be classified into two groups: qualitative and quantitative methods.

Qualitative methods are mathematical procedures that aim to reveal the relationship between easily obtained variables and the variables/properties of interest. Essentially, these methods provide an exploratory step to characterize and sort samples according to their inherent properties. These methods also provide the visualization of data in the form of graphs and plots, which facilitates the discovering of relating/opposing relations, trends, and groups. Another key aspect is the indication of relevant variables to describe the main differences/similarities of variables in the study and also the exclusion variables of no interest [73]. Some of the main qualitative chemometrics methods are Principal Component Analysis (PCA), Linear Discriminant Analysis (LDA) and Supervised Vector Machines (SVM).

The PCA is one of the most widely used methods to obtain qualitative information from datasets. PCA is a method to reduce the large number of variables of the original dataset into a subspace (composed with a reduced number of dimensions) and preserve the variation in data as much as possible. This subspace is composed of few principal components (independent of each other) that aims to explain most of dataset variation. Essentially, data is transformed into coordinates and direction in this new subspace, which gives a visual representation of trends, groups, or the structure of data. The PCA is considered as one of the most widely applied multivariate methods in exploratory data analysis [73,74].

A method widely applied in qualitative evaluation of datasets is LDA. This method aims to obtain a projection of means from known groups in a subspace by maximizing the variance between defined groups and minimizing intergroup variance. The large number of variables is reduced to few new dimensions named canonical functions [74,75]. Essentially, the complex dataset with labeled data is projected in a linear function that maximizes separation of projections of the known groups and minimizes their intergroup separation [76]. Moreover, the Discriminant Analysis can be applied to non-linear and multi-class conditions with proper modifications in the mathematical procedure [75]. It is relevant to remember that PCA and LDA differs in terms of data labeling. Supervised methods (such as LDA) contain data, whereas unsupervised methods (such as PCA) is composed of non-labeled data [74].

Another relevant option is the use of SVM. The SVM is a method that aims to obtain a separating hyperplane with the maximum separation between two groups of datasets. This maximization is the consequence of defining the margin and the supporting vectors. The margin is the distance between the hyperplane and supporting vectors. The supporting vectors are specific values from both datasets that are critical to define the position and orientation of the hyperplane, which is determined to maximize the margin [77,78].

SVM was originally designed for datasets that are clearly separated and without outliers. In cases that a linear hyperplane cannot be obtained due to these effects, a soft margin is necessary to allow some data to be misclassified and achieve the original goal [77]. Another aspect is the use of SVM in datasets that are not separable, and the use of a soft margin does not assist in finding the hyperplane. In this condition, the use of a kernel function is necessary to add an additional dimension to the space of datasets. Essentially, data is projected to a high dimensional space through a defined function and mapping [77].

The evaluation of complex data to obtain relevant information from datasets can also be carried out using artificial neural network (ANN). The ANN can be described as a model composed of several interconnected nodes containing activation functions that receive inputs, process/compute, and send outputs to the next node. Essentially, the harmonious combination of functioning nodes processing information permits the resolution of problems from complex and large datasets [79,80].

ANN can recognize patterns, classify, and cluster datasets as well as give predictions. These applications are attributed to the training of ANN in the dataset, which consist of training ANN to identify hidden patterns to define the weights of nodes and obtain an accurate analysis of the dataset [79]. The development of ANN, especially using parallel architecture (network of interconnected hidden nodes located between the input node and the output node), has advanced to reduce computational time and give fast outputs, which facilitates the progression towards future real-time applications in food processing [81].

Chemometric analysis also has quantitative methods that can succeed in the qualitative evaluation of data (indicated above). Different than qualitative methods, quantitative procedures aim to create mathematical models from the dataset and formulate/test hypothesis. Consequently, unknown samples can be evaluated and classified according to proposed quantitative method [73]. One of the most common procedures to generate mathematical models in chemometrics is the Partial Least Squares Regression (PLSR) method. The PLSR method aims to determine regression models between sets of variables. The method decomposes the data from matrices x (independent variables) and y (dependent variable) into latent variables (vectors from the projection of variables). These latent variables are projected to obtain the maximum covariance between data from x matrix and their related response from y matrix [73,82].

## 7. Application of E-Nose, E-Eye, and E-Tongue

### 7.1. Fresh, Refrigerated and Frozen Meat

The aroma of meat is composed of a complex combination of volatile compounds (also known as odor-active compounds, OAC) that stimulate the olfactory system. In terms of application of E-nose systems in the evaluation of different factors that affect the aroma of meat and meat products, some key aspects have been studied in the last years: presence of pathogenic microorganisms, quality grading, adulteration, and monitoring quality decay during shelf life (Table 1).

The early detection of pathogenic microorganisms in meat and meat products is an important safety measure to prevent foodborne disease outbreak. In this sense, some studies have explored this potential application of E-nose. One relevant example of this application is the detection of *Salmonella typhimurium* in fresh pork using E-nose technology [57]. This study revealed that the accuracy to predict the presence of this bacterium by SVM model was affected by the kernel function. The genetic algorithm had the highest prediction accuracy (r^2^ = 0.989) in comparison to particle swarm optimization and grid search kernel functions (r^2^ = 0.986 and 0.966, respectively). The further examination of the calibrated SVM model (genetic algorithm as kernel function) in independent contaminated pork (known contamination level of 2, 4, and 7 log CFU/g) revealed an elevated accuracy (r^2^ = 0.966) for correct prediction of contamination.

A further experiment in pork meat explored the use of E-nose (PEN3 nose equipment) to discriminate the inactivation of *Salmonella typhimurium* and *Escherichia coli* in terms of ultrasound treatment (20 kHz for 10, 20, or 30 min) in pork [58]. PCA and LDA models clearly discriminated the samples, regardless of ultrasound treatment or bacterium. Moreover, PLSR was selected for quantitative analysis, which led to higher prediction accuracy for *E. coli* (r^2^ = 0.953) than for *S. typhimurium* (r^2^ = 0.937). The further application of the calibrated PLSR model showed the same trend regarding these two bacteria (r^2^ = 0.932 and 0.912 for *E. coli* and *S. typhimurium*). From these two studies (same E-nose model; PEN3 nose), it seems reasonable to infer that kernel function of the SVM model and the type of microorganism can affect the performance of the predictive model and must be considered as key factors.

Some recent studies with E-nose technology indicate variable capacity to differentiate meat stored in different conditions. In the case of chicken meat, a related experiment indicate accuracies of fresh and frozen-thawed superior to 94% for correct classification of both groups [1]. Adulteration is another key aspect related to the quality of meat and meat products. Regulations related to meat and meat products’ authenticity also involve the legal definition and related terms to apply specific legislation for commercialization of meat and meat products in the European Union. The applied regulations cover many aspects such as the Regulation (EC) No 853/2004 for the definition of ‘meat’, ‘mechanically separated meat’, ‘meat preparations’ [83]; Regulation (EU) No 1169/2011 for the definition of minced meat and maximum limits for fat and collagen contents, indication of species, frozen date and location of origin in the label of the foods, the maximum fat and connective tissue levels in food products with meat, and [84]; Regulation (EU) No 1308/2013 for the definition of “sale description” (the name a meat or meat product is sold) [85]; and Regulation (EC) No 1333/2008 for authorized food additives and their legal limits in meat products [86]. Additionally, the Regulation (EU) No 1308/2013 is also related to measures concerning animal diseases and loss of consumer confidence due to public, animal or plant health risks [85].

The utilization of E-nose technology in the detection of adulteration of fresh meat was evaluated in mutton meat artificially adulterated with duck meat [59]. The E-nose system could distinguish different levels of duck meat addition with accuracy in the range of 83–100% for adulteration between 10 and 100%. It is relevant to mention that some overlapping in samples adulterated with 50% and 70% of duck meat would not be entirely distinguishable from each other. Regarding the evaluation during quality decay, some studies indicate reduced capacity to represent an accurate measurement of meat quality state during shelf life by monitoring specific volatile compounds. This outcome was reported for the monitoring of biogenic amine formation in pork tenderloin [87] and total volatile basic nitrogen levels in chicken breast [88]. A related study carried out by Han et al. [89] explored the use of an economic E-nose system to detect the adulteration of beef with duck meat. The authors obtained a correct identification rate of 83.3% (samples composed of duck, duck and beef combinations, and beef meat). Interestingly, the study also revealed that combining the low-cost E-nose with near infrared Fourier transform-near-infrared spectroscopy data improved prediction capacity of the system (reducing root means square error and improving the correlation coefficient).

The use of E-eye in the evaluation of quality has been studied in fresh meat from different species and many meat products. The evaluation of quality with E-eye has been targeting main applications in terms of color, marbling level, quality prediction, detection of defects, and sorting operation (Table 1). One of the key attributes evaluated in meat and meat products is their color. Special emphasis is given to the relation between color and perception of freshness. In case a meat cut does not have the characteristic/expected color, it is likely to be discarded or not be purchased [90]. In this sense, some studies compared the performance of E-eye with colorimeters and reported good correlation between E-eye and colorimetric data on chicken meat [91], particularly for lightness, whereas reasonable regression coefficients were obtained for redness and yellowness (L*a*b* space). 

**Table 1:** Application of E-nose, E-eye, and E-tongue in fresh meat.

Meat	Application	Equipment Model, Manufacturer, and Number of Sensors	Data Treatment	Main Outcomes	Ref.
Applications of E-nose					
Pork (*longissimus*)	Detection of *Salmonella typhimurium*	PEN3 nose (Airsense, Schwerin, Germany); 10 metal-oxide semiconductor sensors	(q): PCA; (Q): SVMR and metaheuristic optimization algorithms (GA, GS, and PSO)	Clear separation of treatment in regions of PCA; model had elevated accuracy to predict the presence of *Salmonella typhimurium* with GA-SVMR (r^2^ = 0.989) followed by PSO-SVMR (r^2^ = 0.986)	[57]
Pork (*longissimus*)	Modeling the reduction in *Salmonella typhimurium* and *Escherichia coli* with US treatment (20 kHz for 10, 20, or 30 min)	PEN3 nose (Airsense, Germany); 10 metal-oxide semiconductor sensors	(q): PCA and LDA; (Q): PLSR	Clear separation of treatment in regions of PCA and LDA; accuracy of model to predict the bacterial reduction from US treatment was elevated for both microorganisms: *Salmonella typhimurium* (r^2^ = 0.912) and *Escherichia coli* with (r^2^ = 0.932)	[58]
Chicken breasts and thighs	Differentiate fresh and frozen-thawed samples	Author’s own E-nose system (power supply, controller, cylindrical sample holder, valves, air and vacuum pump, air filter, sensor array, data acquisition card, computer, and software for data management); 8 sensors (for alcohols, CO, CH_4_, C_3_H_8_, C_4_H_10_, SO_2_, and steam of organic solvents)	(q): F-KNN	Model had elevated accuracy for correct classification of fresh (95.2%) or frozen-thawed (94.7%) meat	[1]
Mutton	Detection of adulteration with duck meat (10, 30, 50, 70, and 100%)	PEN3 (Win Muster Air-sense Analytic Inc, provided by AIRSENSE Company, Germany); 10 sensors	(q): LDA; MLPN	LDA indicated clear separation among adulteration levels and small overlap between 50 and 70% of duck meat; model had elevated accuracy (83–100%) for correct classification of each adulteration level	[59]
Pork tenderloin	Quality decay during shelf life (biogenic amine content; 0, 3, 5, and 7 days at 4 °C)	Food Sniffer (ARS.LAB Inc., Redwood City, CA, USA); sensor components n.i.	(q): PCA	System correlated reasonable with *Enterobacteriaceae* counts (r = 0.890); poor correlation between E-nose and biogenic amine content	[87]
Chicken breast	Quality decay during shelf life (TVB-N; 0, 1, 2, 3, 4, and 5 days at 4 °C)	Author’s own E-nose system (data acquisition, modulating, and transmitting unit; gas sensor array and chamber system; and power and gas supply unit); 8 sensors	(q): PCA	PCA had poor discrimination capacity based in TVB-N content	[88]
Applications of E-eye					
Chicken breast	Color evaluation	Doc L-Pix image system (Loccus Biotecnologia, Brazil); color system: RGB, XYZ and L*a*b*	(Q): LRA	High correlation between computer vision system and colorimeter measurement for L* (r^2^ = 0.99); limited correlation for a* (r^2^ = 0.74) and b* (r^2^ = 0.88)	[91]
Center cut pork loin	Color evaluation	Digital camera (MV-VS141FM/C, Micro-vision Ltd., China); color system: RGB, HIS, and L*a*b*	(Q): LRA	Computer vision was suitable for color evaluation; correlation of E-eye with colorimeter was dependent of color space; highest correlation was obtained using L*a*b* space (r^2^ = 0.83)	[61]
Pork (*longissimus thoracis et lumborum*)	Color evaluation and grading for color	CCD camera and 2 bars with white LED; color system: RGB, HIS, and L*a*b*	(Q): PLSR and SVMR	SVMR was more accurate (73.4%) to correctly predict color grade than PLSR (68.3%) method (data from three color systems)	[62]
Pork and beef (*longissimus thoracis*)	Marbling classification	Single lens reflex camera, model Nikon SLR D7000 (Nikon Co. Ltd., Tokyo, Japan); color system: HSL	(q): K-NN	High accuracy for grading (81.6 and 76.1% in bovine and swine meat, respectively)	[64]
Center cut pork loin	Intramuscular fat content	CCD camera, two white LED bar lights, and Computer; color system: RGB, HIS, and L*a*b*	(Q): LRA, stepwise regression model, and SVMR	SVM had higher overall accuracy (75%) than stepwise regression model (63%) to estimate pork intramuscular fat (data from three color systems)	[68]
Pork loin	Color and marbling	Industrial camera (NI 1776C smart camera, National Instrument, Ltd., Austin, TX, USA) with a 1/1.8” F1.6/4.4–11-mm lens (LMVZ4411, Kowa, Ltd., Tokyo, Japan), a 44-in. dome light (DL180, advance illumination, Ltd., Rochester, VT, USA); color system: L*a*b*	(Q): LRA and SVMR	High prediction accuracy for color score based in L* values (92.5%) and marbling score (75.0%); LRA established poor correlations for color score (r^2^ = 0.64) with sensory data and marbling grade (r^2^ = 0.54) with intramuscular fat content	[63]
Chicken breast (*pectoralis major*)	Classification according with wooden breast myopathy	Doc L-Pix image system (Loccus Biotecnologia, Brazil); color system: HSV	(q): SVM, MLP, J48 DT	The use of SVM reach accuracy of 91.8% to correctly classify samples	[65]
Pork (*longissimus lumborum*)	Identification of defects	CANON EOS 350D with an EF-S 60-mm macro lens digital camera; color system: RGB, HSV, and HSL	(Q): LRA	High accuracy to with HSL system to detect PSE (91%) and DFD (73%); low capacity to differentiate red, soft, and exudative from red, firm, normal	[92]
Pork (m. *semimembranosus*)	Identification of defects-classification	Digital camera Canon EOS 350D with the lens EF-S 60 mm; color system: RGB, HSV, and HSL	(Q): LRA	Low accuracy	[93]
Chicken breast, leg, fillet, drumstick, and wing	Sorting cuts	Digital CCD camera (ace1300-200uc, Basler, Germany) and linear light-emitting diode (LED) tubes; color system: HSV and RGB	(q): PLS, LDA, and ANN	Prediction accuracy 96% at continuous measuring	[67]
Applications of E-tongue					
Cattle and buffalo meat (*longissimus*)	Discriminate cattle breeds	Model n.i. (Alpha M.O.S., Toulouse, France); 7 sensors	(q): LDA	Separation in three groups: Angus, Hungarian grey, and cluster composed of other breeds	[44]
Mutton	Detection of adulteration with pork or chicken meat (0, 20, 40, 60, 80, and 100%)	α-Astree (Alpha M.O.S, Toulouse, France); 7 sensors	(q): CDA	Elevated accuracy to discriminate pork (100%) and chicken (80–90%) adulteration, regardless of adulteration level	[94]
Beef (*semitendinous*)	Discrimination according to irradiation level (0, 1.5, 3.0, and 4.5 kGy)	α-Astree 2 E-tongue (Alpha M.O.S., Toulouse, France); 7 sensors	(q): PCA	Clear separation of samples according to irradiation dose	[95]
Beef	Quality decay during shelf life (ammonia and putrescine; 1, 2, 3, 4, 5, 6, 7, 8, 9, and 10 days at 4 °C)	Author’s own E-tongue system (sensor, potentiostat/galvanostat, and computer); 3 electrodes individually coated with modified carbon screen-print, Ag and carbon	(q): PCA and PLS-DA	Differentiation of samples in four groups: days 1-2, 3-4, 5–7, and 8–10; correlation coefficient between 0.95 and 0.98 during shelf life	[70]

ACO: Ant Colony Optimization; ANN: Artificial Neural Network; CDA: Canonical Discriminant Analysis; DFD: Dark, firm, and dry; DT: Decision Trees; F-KNN: Fuzzy K-Nearest Neighbors algorithm; GA: Genetic Algorithm; GS: Grid Searching; KNN: K-Nearest Neighbors algorithm; LDA: Linear Discriminant Analysis; LRA: Linear Regression Analysis; MLP: Multilayer Perceptron; MLPN: Multilayer Perceptron Neural Networks Analysis; PCA: Principal Component Analysis; PLS: Partial Least Squares; PLSR: Partial Least Squares Regression; PSE: Pale, soft, and exudative; PSO: Particle Swarm Optimization; SVM: Support Vector Machine; SVMR: Support Vector Machine Regression; VCPA: Variable Combination Population Analysis. (Q): Quantitative or calibration methods, (q) qualitative or classification methods. n.i.: not indicated.

A similar outcome was reported for the use of E-eye in the color analysis of fresh pork [61]. The use of L*a*b* space provided the highest correlation coefficient in relation to other color spaces. It also relevant to mention that in the evaluation of color, the selection of the chemometric method can also affect the quality of prediction. This outcome was reported in a recent study with fresh pork where SVMR provided more accurate prediction than PLSR for color evaluation and matching to a grading system [62]. These studies highlight the importance of selecting the appropriate color space and chemometric method to improve the correlation between colorimetric and E-eye data as well as increase prediction accuracy.

Marbling is another key factor for purchase among consumers. The distribution and amount of intramuscular fat in meat is known to affect the sensory attributes of tenderness, juiciness, and flavor [96]. Due to the possibility to grade marbling using visual appraisal, the use of E-eye has been tested in fresh meat. Recent experiments in fresh beef and pork indicated that an elevated degree of accuracy (81.6 and 76.1% in beef and pork, respectively) can be obtained using this technology [64]. The selection of data treatment has been indicated to affect the accuracy of the method for meat marbling grading [68]. The use of SVM provided a system with higher precision than using stepwise regression model to treat E-eye data obtained from raw pork.

A more comprehensive use of E-eye technology in the evaluation of meat quality has also been explored in recent studies and considered in the prediction of physicochemical characteristics and sensory attributes. Although this application can provide a more realistic evaluation of the meat quality, E-eye systems seem to display different accuracies among variables. This outcome was reported in a recent experiment to evaluate color and marbling grade in pork meat when data samples were obtained from different meat processing plants [63]. The accuracy for correct color grading was higher than that obtained for correct marbling grade (92.5 vs. 75.0%, respectively) using SVM for data treatment.

The detection of defects that affect quality is another interesting application of E-eye technology. One major quality aspect of poultry is the occurrence of white stripes in the pectoralis major muscle that affect the visual aspect, physicochemical properties and nutritional characteristics [97]. In this case, a study evaluated the accuracy of different algorithms (SVM, Multilayer Perceptron, Random Forest, and J48) to distinguish normal chicken breast from wooden breasts [65]. The SVM method had the highest accuracy (91.8%) in comparison to other methods (90.67, 87.83, and 85.25% for Multilayer Perceptron, Random Forest, and J48, respectively).

Another relevant meat defect found in meat is the occurrence of pale, soft, and exudative characteristics that affect the eating quality, especially for pork [98]. The identification of PSE pork with E-eye system was tested in *longissimus lumborum* muscle from pork and indicated an elevated capacity to detect normal from PSE samples with 80% accuracy [92]. However, the use of an E-eye system in pork *semimembranosus* muscle did not achieve the same accuracy. One of the possible reasons for this outcome was the high variability of surface color [93].

Additionally, the sorting of meat cuts is another operation that could be carried out using E-eye technology. In this case, a recent experiment by Teimouri et al. [67] with chicken cuts (breast, leg, fillet, drumstick, and wing) transported in a conveyor operating at two velocities (0.1 and 0.2 m/s). These authors compared the overall accuracy of PLSR and LDA algorithms and observed that LDA was more accurate to correctly classify each cut than PLSR for data validation (96% and 89% for LDA and PLSR, respectively). When the velocity of the conveyor was considered, the authors indicated that overall accuracy was affected (94% and 93% for 0.1 and 0.2 m/s, respectively). Consequently, operating the conveyor at 0.1 m/ would result in the highest accuracy for correct classification of cut. This study is an important example of the potential advantages that E-eye can bring to the recent trends in automation and online control in food production [10].

The E-tongue has also been studied to determine several aspects related to quality and shelf life of fresh meat. One example is the identification of cattle and buffalo meat according to animal breed [44]. In this case, samples from Angus and Hungarian grey breed were clearly separated from the other breeds (domestic buffalo, Hungarian Spotted cattle, and Holstein), indicating a potential application to ensure the traceability for these two breeds. Another relevant line of study is the detection of meat adulteration wherein a recent experiment indicated that E-tongue showed elevated accuracy to identify the different levels of adulteration of mutton with pork [94]. This experiment also indicated that E-tongue technology could correctly identify mutton samples containing different levels of chicken meat, but with a lower accuracy obtained from pork adulteration. 

The E-tongue system was also tested to discern irradiation treatments in beef samples [95]. The system could differentiate treatments according to irradiation treatment and posterior evaluation of flavor characterization to indicate the association of 0 and 1.5 kGy with umami, sweetness, saltiness and bitterness, and the association of 3 kGy with sourness, whereas 4.5 kGy was characterized by the degradation of flavor descriptors. In another experiment with E-tongue technology with sensors for ammonia and putrescine, this technology showed capacity to monitor the quality decay in refrigerated beef [70].

### 7.2. Processed Meat and Meat Products

The evaluation of meat products using E-nose, E-eye, and E-tongue has been reported in several studies (Table 2). Similarly, to observe for fresh meat, the use of E-nose has also been considered for the detection of pathogenic microorganisms in meat products. One example is the detection of ochratoxin A-producing *Penicillium* in dry-cured sausage [99]. The E-nose system achieved 88% of accuracy to identify (at strain level) samples contaminated with *Penicillium* capable of producing ochratoxin A. Quality grading is another potential application for E-nose that has been studied in the last few years. In braised pork, the use of E-nose was tested to differentiate geographical origin of the meat in China (Beijing, Changsha, Chengdu, Guangzhou, Hangzhou, Jinan, Meizhou, Shanghai, Shenyang, Shijiangzhuang, Wuxi, and Xiamen) [100]. The PCA generated from E-nose data varied depending on the type of sample wherein the separation of samples according to their origin was partially achieved from lean meat. Conversely, PCA did not provided a clear separation of samples produced in different locations from fat fraction, which suggested that lean meat would be a better fraction to collect samples to distinguish braised according to the origin. A similar experiment was carried out to grade the quality of dry-cured hams using E-nose [60]. In this case, the separation of groups according to the quality grade was achieved using PCA, and posterior generation of a regression model (especially with the SVM method) achieved high accuracy in a short processing time.

The detection of adulteration with soy protein in emulsion-type meat products has also been studied with E-nose [101]. In this case, separation of control from samples containing soy protein was possible, but samples containing 20% and 30% of soy protein would be identified as similar. Since a linear chemometric method did not provide sufficient separation regarding the level of soy protein, the probabilistic neural network method was applied to compare the extracted features (area values vs. maximum response values). This strategy indicated that maximum response values was a more suitable data source than area values (reliability of classification of 96% and 94%, respectively) to separate the groups of samples in terms of soy protein content. This outcome suggests that the selection of measured variables to extract valuable information can affect the reliability of the classification method.

Processing conditions are directly related to the quality of meat products. For instance, the E-nose technology has proven effective to differentiate chicken meat stewed for 1 h from samples prepared after 2 or 3 h [102]. Another related experiment tested the accuracy of E-nose technology to differentiate braised chicken prepared with different conditions [103]. In this case, it was possible to distinguish samples according to thermal treatment (untreated, cooked at 84 and 95 °C, and sterilized) and storage time. Specifically, the samples cooked at 84 and 95 °C were grouped together with fresh samples. This outcome indicates that these two thermal treatments had better preservation effects in the volatile compounds associated with fresh samples. Conversely, applying sterilizing conditions resulted in the modification of volatile composition in relation to freshness and samples cooked at 84 and 95 °C.

E-nose systems have also been used to monitor the formation of OAC in sugar-smoked chicken processing [104]. It is worth commenting that, in this case, the evaluation of skin samples during processing has proven to be more accurate to distinguish processing stages. In the PCA plot, the fat samples obtained from different processing stages could be clearly separated from each other, whereas overlapping of groups was observed in samples obtained from chicken breast.

The decay of quality during shelf life can also be monitored using E-nose systems. A recent experiment with bacon indicated that E-nose technology could be used to distinguish samples according to the period of storage [105]. In this case, the E-nose with PCA was suitable to indicate which samples were stored for 22, 30, or 45 days. Conversely, bacon stored for 0, 7, and 15 days were not clearly separated in the PCA plot, suggesting that up to 15 days bacon had similar volatile composition.

The use of E-eye technology can also be applied to monitor the changes in superficial color of meat processing (Table 2). This application was tested in the production of smoked chicken thighs, which had accuracy of color determination superior to 95% using K means algorithm for data treatment [106]. Moreover, the E-eye technology also assisted in the validation of a card system to evaluate the color of samples. The use of K-mean methods was relevant to produce regression models to determine the color in terms of smoking time, which can be seen as a potential strategy to improve the quality control in the meat industry. Likewise, another study reported the evaluation of intramuscular fat content in dry-cured ham has also been studied using E-eye technology [69]. This system used a convolutional neural network to achieve high accuracy and precision in fat identification without sample destruction.

Meat products can also be sorted using an E-eye system [66]. Accuracy in correct identification of meat products (smoked meats, sausages and meat blocks) ranged between 83 and 100%. The system also showed capacity to predict chemical composition with coefficients of correlation in the range of 0.70–0.92. Another related experiment reported the discrimination of Chinese dry-cured hams according to their geographical origin and quality grade [107]. In this case, the sorting of samples includes both image and sensory analysis data in the PCA and Cluster analysis. Both chemometric methods provide similar outcomes to classify the samples in terms of origin and quality grade, but only three groups were formed among the nine different hams.

Many studies aim to explore the application of E-tongue to evaluate the quality of processed meat and meat products. One relevant example is the discrimination of sous-vide-cooked beef in terms of temperature (especially 45 and 60 vs. 70 °C) [108]. Moreover, the treatment was also associated with flavor; cooking at 60 °C was associated with astringency, whereas cooking at 70 °C was related to umami, richness and bitterness. A related study explored the characterization of E-tongue in the changes promoted by braising cycles in pork broth [109]. The modification in broth characteristics were monitored with E-tongue technology, wherein the samples that could be classified according to the number of cycles were observed mainly in those containing meat (meat + herbs and only meat) rather than only seasoning in the composition of broth.

## 8. Advantages and Disadvantages

Since the use of electronic technologies mimicking human senses implies a change in the current perspective of quality evaluation, it is relevant to comment on the important advantages and disadvantages of these systems. Essentially, these systems are characterized by the absence of sensory fatigue, short analysis time per sample, easy training, and operation without high-skilled operators [27,34,110,111].

In the case of E-nose, the main advantages are related to each sensor. For instance, metal oxide semiconductor sensors have sensitiveness as low as 1 ppm to key flavor active compounds. Another relevant sensor is composed of conducting polymers that can be fabricated to detect specific flavor-active compounds, can have a detection threshold down to 10 ppm, and consume less energy in comparison to metal oxide semiconductor sensors. However, these sensors have some disadvantages such as: the necessity to operate at high temperature (up to 400 °C), control of experimental conditions (humidity, pressure, temperature, and gas velocity), and relative low number of sensors in relation to the human nose [27]. In other words, the E-nose equipment must be properly prepared in advance by the heating of the sensor and gas flow (long preparation time and intense energy consumption to achieve and sustain optimum operational temperature). The use of an airtight vial under constant temperature for a known period is also necessary to standardize the protocol and reduce variations in the composition of headspace.

Exposure to humidity (water poisoning) with eventual obstruction of active sites and generation of electrons is a major interference for oxide semiconductor sensors, leading to limited sensibility. Reducing water poisoning can be achieved by incorporating dopants and additives (such as CeO_2_, NiO, Pb, and Tb) in the sensor. However, the sensor performance can be affected due to alteration of sensor sensitiveness to gases or increased resistance [27,112]. Consequently, advances in the metal oxide semiconductor are still necessary. Sensor drift is another key aspect related to the performance of E-nose. Drift can be defined as the temporal fluctuation in sensor response to the presence of specific volatile compounds and is caused by different factors such as aging and contamination [113,114].

The E-eye is another technology with interesting advantages such as non-destruction of samples, easy operation, non-invasiveness and low hazardous aspect, minimal or no sample preparation, and acquisition and permanent storage of high-resolution images. In terms of disadvantages, this tool requires a controlled environment with consistent light exposure in a dark space to avoid interference from external sources of light, can only evaluate one side of samples per time, requires special attention to the separation of background for correct data acquisition, and needs constant calibration [34,111]. One central aspect in the handling of meat samples for E-eye measurements is the necessity to use samples that meet the known criteria related to the expected application of this electronic system. For instance, the analysis of marble grading relays in the training of the system with previously known samples that meet the different levels of marbling in fresh meat [64] or the use of a trained specialist that can effectively identify and differentiate chicken with wooden breast myopathy from normal chicken breast [65].

Finally, the main advantages of E-tongue are the simplicity (especially with impedance-based sensors), long-term stability (particularly for optical mass-based sensors), customization for specific compounds (principally for potentiometric sensors), and possibility to evaluate and test foods containing toxic compounds (mycotoxins, for instance). The main disadvantages of E-tongue are the pre-treatment of samples (especially for solid foods such as meat and meat products) and short lifetime of sensor components due to absorption of food components (particularly for potentiometric sensors) [27,110]. 

From a comprehensive point of view, some issues must be considered when working with any electronic systems mimicking human senses. One of the issues is the lack of consensus about the mathematical models to determine key analytical indicators, especially the limit of detection and sensitivity. This concern is related to absence of proven evidence that a low concentration can actually cause a distinguishable signal from a blank and the arbitrariness of the value of standard deviation (from 2 to 10) to estimate the limit of detection [115]. It is also relevant to comment that reproducibility and repeatability of both E-nose and E-tongue is dependent of the number of samples. Consequently, multiple samples (*n* > 10) are usually necessary to have systems with high accuracy and precision, especially during training and validation of models [27].

Although important advances have been achieved to evolve E-nose, E-eyes, and E-tongue towards more precise and accurate applications, the individual use of these electronic systems is not comprehensive enough to simulate the human senses to judge the quality of meat and meat products. It is relevant to remember that human perception of meat and meat products’ quality comprises the combination and the simultaneous processing of stimuli acquired from eyes, nose, and tongue. In this sense, integrating/fusing the datasets of E-nose, E-eyes, and E-tongue is a natural progression to the automation of quality evaluation in meat and meat products production and quality assessment. The advantage of fusing data is the probability of correct classification and prediction of quality, but the new concerns raised lead to additional challenges in terms of data quality (especially information preservation), computational time, and applicability in real food processing conditions [116].

## 9. Conclusions

The electronic systems mimicking the senses of smell, vision, and taste have been gaining attention from researchers, and their applications have expanded to assist in the evaluation of quality and shelf life of meat and meat products. The main strength of E-nose, E-eye, and E-tongue are their versatility to characterize and discriminate the application of preservation techniques (chilling, frozen, irradiation, for instance), detect adulteration (such as meat from different species and vegetable proteins), quality grading (marbling), monitor the quality decay during shelf life, and indicate the presence of toxic microorganisms and compounds without complex, long-lasting, laborious and human-based methods.

This promising scenario is also characterized by the necessity to promote more studies about these three technologies in the area of meat and meat products. The industrial sector is currently evolving in the context of the 4th industrial revolution that is characterized by the intensification of automation and online monitoring. The electronic systems discussed in this review have a crucial role by providing reliable, fast, low-cost, portable options to evolve current production lines and gain competitiveness in the globalized food market. However, advances are still necessary. Most of the studies provide results at laboratory scale, and little is known about the conditions that mimic, at least in part, conditions found in the meat industry with conveyors and continuous production conditions. It is important to mention that variations exist in the developmental stage among these technologies. E-nose and E-tongue equipment are commercialized technologies, whereas E-eye systems are still largely composed of equipment designed in the laboratories, which indicates the necessity to progress the technological development of E-eye to commercial applications.

Additionally, it is also relevant that the progression in the development of E-nose, E-eye, and E-tongue be aligned with current developments in the field of meat and meat products. Current studies have explored the use of electronic systems in commercial meat (widely consumed meats: pork, beef, and chicken) and meat products (traditional processing, practices, and formulations), and limited knowledge has been generated around global challenges changing the production of meat and the development of meat products.

The development of sustainable meat production systems is gaining more attention from consumers that choose the consumption of meat produced with environmentally friendly practices; animal feed production, allowing animals to graze, and proper animal care (animal welfare) can be cited as relevant aspects. The evaluation of meat produced from these systems has not been characterized by electronic systems mimicking human senses and little information is known about the challenges associated with samples and dataset pretreatment or Chemometric analysis from meat produced from sustainable systems. It is important to remember that animal diet and feed composition can affect meat quality (in terms of composition, shelf life, and sensory attributes).

The development of sustainable actions is also related to the production of meat products. Food ingredients and nutrients (especially sodium chloride, nitrate and nitrite salt, and fat) are known to have a direct effect on human health by preserving health status and also negatively affecting health by increasing the risk of severe diseases such as obesity, cardiovascular diseases, and cancer. The reformulation of meat products has been gaining the attention of consumers, governmental agencies, and companies in the food sector. Reducing and replacing sodium, saturated fat and nitrate/nitrite salts from commercial sources are among the most widely studied strategies in the food science area. Functional meat products have also been studied, and important advances in the use of prebiotic ingredients, probiotic strains, and bioactive phytochemicals are among the main options explored by food scientists.

Another aspect related to meat production and meat products processing is the emerging interest in alternative dietary proteins. Meat production systems have been criticized due to the generation of residues, greenhouse gas production, and deforestation during the last decade. Consequently, alternative sources of proteins to partially replace animal proteins in meat products became an emerging central topic of research. The studies exploring the effect of partially replacing animal protein with vegetal sources are accumulating, and those from insects are gaining more attention. Although much information has been generated in the last decade, the use of E-nose, E-eye, and E-tongue in reformulated meat products, the influence and aspect of each remain insufficiently explored.

## Figures and Tables

**Table 2:** Application of E-nose, E-eye, and E-tongue in processed meat and meat products.

Meat Product	Application	Equipment Model, Manufacturer, and Number of Sensors	Data Treatment	Main Outcomes	Ref.
Applications of E-nose					
Dry-cured sausage	Detection of ochratoxin A-producing strains of *Penicillium* sp.	ISE Nose 2000 (SoaTec S.r.l., Parma, Italy), 12 metal-oxide semiconductor sensors	(q): LDA	LDA of E-nose data separated samples containing *Penicillium* at strain level; elevated accuracy for identification of samples containing ochratoxin A-producing strains (88%)	[99]
Braised pork	Discrimination according to geographical origin	PEN3 E-nose (Airsense Analytics GmBh, Germany); 10 metal-oxide semiconductor sensors	(q): PCA	Better separation according to location using lean meat (overlap for half of groups) fraction than fat (overlap of almost all groups) fraction of samples	[100]
Dry-cured ham	Quality grading (first, second and third grade)	Author’s own E-nose system (sensor chamber, control module, and wireless communication module); 12 sensors (for acetone, ammonia, butane, ethane, ethanol, hydrogen, hydrothion, isobutene, methanol, methane, methanthiol, methylbenzene, propane, trimethyl amine, and vapors of organic solvents)	(q): PCA and T-SNE; (Q) SVM, KNN, and LR	Clearest separation of samples according to grades was possible using with either PCA or T-SNE with the optimization with MIME-(SVM-BFECV) protocol; model had high accuracy (99.5%) and fast processing time (15.7 s); accuracy reduced and processing time increased in second and third grade samples	[60]
Pasteurized sausage	Detection of adulteration with soy protein (10, 20, and 30%)	Author’s own E-nose system (compressor filter with a silica gel, sealed chamber with sensors, frequency monitoring system, and computer); 7 sensors individually coated with dicyclohexano-18-crown-6, poly(ethylene glycol) 2000, poly(ethylene glycol adipate), poly(ethylene glycol sebacate), poly(diethylene glycol succinate), Triton X-100, and polyvinylpyrrolidone	(q): PCA; PNN	PCA indicated maximum response values as relevant variable to separate groups, but poor separation was obtained from samples with 20% and 30% of soy protein; elevated accuracy for correct classification of 0, 10, 20 and 30% soy protein in sausage (96%) using non-linear strategy (PNN)	[101]
Chicken stew	Evaluate the effect of stewing time (1, 2, and 3 at 95–99 °C)	FOX 4000 (Alpha M.O.S., Toulouse, France); 18 metal oxide semiconductor sensors	(q): PCA	Clear separation of samples with different times of stewing, especially 1 h samples from 2 and 3 h samples	[102]
Dezhou-braised chicken	Intensity of thermal treatment (84 °C for 35 min, 95 °C for 30 min, and 121 °C for 20 min) and quality decay during shelf life (0, 7, 15, 22, 30, and 45 days at 4 °C)	FOX 4000 (Alpha M.O.S., Toulouse, France); 18 metal oxide semiconductor sensors	(q): PCA	Separation in three main groups: (1) control after 30 and 60 days, (2) 84 and 95 °C at any storage time and fresh sample, and (3) sterilized samples at any storage time	[103]
Sugar-smoked breast and skin chicken	Characterize the effect of processing stages (pickling, air-drying, baking, and sugar-smoking) in flavor accumulation in breast and skin	PEN 3.5 E-nose (Airsense Analytics GmBh, Germany); 10 metal-oxidesemiconductor sensors	(q): PCA	Discrimination of processing stages was clearer for skin than breast (overlap of processing stage groups) samples	[104]
Bacon	Quality decay during shelf life (0, 7, 15, 22, 30, and 45 days at 4 °C)	Fox 4000 (Alpha M.O.S., Toulouse, France); 18 metal oxide semiconductor sensors	(q): PCA	Separation in four groups: up to 15 days (overlap of groups), 22, 30, and 45 days	[105]
Applications of E-eye					
Smoked chicken thighs	Color and sensory analysis	EOS-M5, Canon, Tokyo, Japan; color system: RGB and L*a*b*	(q): K-means algorithm; (Q): LRA	Production of colorimetric cards for color evaluation; high prediction accuracy of K-mean (r^2^ = 0.995) and K-mean + noise (r^2^ = 0.952) reduction model for smoking time as function of RGB space	[106]
Dry-cured ham	Intramuscular fat content	Digital camera (Canon EOS 50D); color system: RGB	(Q): SVM	Development of a convolutional neural network to identify intramuscular fat, accuracy of 99% and precision of 84% for intramuscular fat identification	[69]
Meat products	Color, chemical composition and texture	Epson Perfection 4490 Photo flatbed scanner; color system: R, G, B; X, Y, Z Lab* and U, V, S	(Q): LRA	Correlation between chemical composition and image texture parameters was 0.7–0.92; high classification accuracy (83–100%)	[66]
Chinese dry-cured hams	Color	IRIS VA 300 computer vision (Alpha M.O.S., Toulouse, France); color system: L*a*b*	(q): PCA and Cluster analysis	Clear separation of 3 groups from PC and Cluster analysis: Jinhua top-class ham; Xuanwei one-year-fermented and two-year-fermented hams; and hams from other origins and grades	[107]
Applications of E-tongue					
Sous-vide cooked beef	Discrimination according to stages (one of two), temperature (45, 60, and 70 °C) and time (3, 6, 9 and 12 h)	SA402B (Insent, Tokyo, Japan), 5 sensors (for detection of sour, bitter, astringent, umami, and salty compounds)	(Q): PLSR	Clear separation of samples according to cooking temperature in three groups: control; 45 and 60 °C, and 70 °C	[108]
Braised pork broth	Effect of braising cycles (up to *n* = 10) in broth (meat + spices; only meat) or soup (only spices)	Astree (Alpha M.O.S, Toulouse, France); 7 sensors	(q): PCA	Separation in groups for meat + spices (8 groups: 1, 2, 3, 4, 5, 6, 7, 8, and 9–10), for only meat (10 groups: 1, 2, 3, 4, 5, 6, 7, 8, 9, and 10), and for only spices (4 groups: 1, 2–5, 6, and 7–10)	[109]

BFECV: Backward Feature Elimination with Cross-Validation; DFA: Discriminant Factor Analysis; KNN: K-Nearest Neighbors algorithm; LR: Logistic Regression; LRA: Linear Regression Analysis; MIME: Mutual Information Mixed Evaluation; PCA: Principal Component Analysis; PLSR: Partial Least Squares Regression; PNN: Probabilistic Neural Network; SVM: Support Vector Machine; and SVMR: Support Vector Machine Regression.

## Data Availability

Not applicable.
